# High Kanamycin Concentration as Another Stress Factor Additional to Temperature to Increase pDNA Production in *E. coli* DH5α Batch and Fed-Batch Cultures

**DOI:** 10.3390/microorganisms7120711

**Published:** 2019-12-17

**Authors:** Fernando Grijalva-Hernández, Jesús Vega-Estrada, Montserrat Escobar-Rosales, Jaime Ortega-López, Ricardo Aguilar-López, Alvaro R. Lara, Ma. del Carmen Montes-Horcasitas

**Affiliations:** 1 Departamento de Biotecnología y Bioingeniería. Centro de Investigación y Estudios Avanzados del Instituto Politécnico Nacional (CINVESTAV-IPN) Av. Instituto Politécnico Nacional No. 2508, Col. San Pedro Zacatenco, México City 07360, Mexico; fheralexgh@gmail.com (F.G.-H.); vegaj@cinvestav.mx (J.V.-E.); montzzze@yahoo.com.mx (M.E.-R.); jortega@cinvestav.mx (J.O.-L.); raguilar@cinvestav.mx (R.A.-L.); 2 Departamento de Procesos y Tecnología, Universidad Autónoma Metropolitana-Cuajimalpa. Av. Vasco de Quiroga 4871, Santa Fe, México City 05348, Mexico; alara@correo.cua.uam.mx

**Keywords:** antibiotic concentration, heat stress, metabolic burden, morphological changes, overflow metabolism, plasmid DNA, high cell density

## Abstract

Plasmid DNA (pDNA) vaccines require high supercoiled-pDNA doses (milligrams) to achieve an adequate immune response. Therefore, processes development to obtain high pDNA yields and productivity is crucial. pDNA production is affected by several factors including culture type, medium composition, and growth conditions. We evaluated the effect of kanamycin concentration and temperature on pDNA production, overflow metabolism (organic acids) and metabolic burden (neomycin phosphotransferase II) in batch and fed-batch cultures of *Escherichia coli* DH5α-pVAX1-NH36. Results indicated that high kanamycin concentration increases the volumetric productivity, volumetric and specific yields of pDNA when batch cultures were carried out at 42 °C, and overflow metabolism reduced but metabolic burden increased. Micrographs taken with a scanning electron microscope (SEM) were analyzed, showing important morphological changes. The high kanamycin concentration (300 mg/L) was evaluated in high cell density culture (50 gDCW/L), which was reached using a fed-batch culture with temperature increase by controlling heating and growth rates. The pDNA volumetric yield and productivity were 759 mg/L and 31.19 mg/L/h, respectively, two-fold greater than the control with a kanamycin concentration of 50 mg/L. A stress-based process simultaneously caused by temperature and high kanamycin concentration can be successfully applied to increase pDNA production.

## 1. Introduction

DNA plasmids (pDNA) are extrachromosomal molecules that replicate independently to the host chromosome but using their own replication machinery [1]. pDNA is used in vaccines and treatment of genetic diseases and cancer. However, clinical trials require doses in the order of milligrams of supercoiled isoform of pDNA (sc-pDNA) which is preferred for use in gene therapy and vaccination because has a superior biological activity compared to other isoforms. Therefore, extensive studies of pDNA production systems are necessary to obtain high pDNA yields and productivity [2,3]. The large-scale production of pDNA presents the same challenges as those of the production of recombinant proteins. But in pDNA production, the priority is to reach maximum sc-pDNA yields using minimum time and resources [4,5]. Until now, *Escherichia coli* DH5α is the most studied commercial strain to improve the pDNA production, although several strains have been developed for the same objective [3,6,7]. pDNA production is affected by host strain, plasmid type and size, genetic modification of the host strain, culture type, medium composition, and growth conditions, such as: the specific growth rate (*µ*); the percentage of dissolved oxygen (%DO); the increase of temperature and heating rate; and the pH control and gradients (revised by Islas-Lugo et al. [8]). Organic acids production (mainly acetic acid) impact cell growth and plasmid yields during the aerobic growth of *E. coli* on glucose, glycerol, and other carbohydrates [9,10]. Higher growth temperatures and temperature up-shift from 35 to 42 °C increase overflow metabolism and reduce biomass yield, but increase specific pDNA yield when the cell contains a pUC temperature inducible replication origin [11,12]. In addition, temperature up-shift causes a heat shock response by synthesizing heat shock proteins (HSPs). These HSPs include proteases and chaperones to reduce problems of misfolded, unfolded or denatured proteins [13].

In recent years, pDNA containing kanamycin resistance gene (*nptII*) and other antibiotic resistance genes have still been used for pDNA production research, even though the current trend is to use antibiotic-free plasmid in pDNA vaccines production due to the risk of serious hypersensitivity reactions in patients [14,15,16]. Kanamycin is not commonly used to treat human infections [17,18,19], and *nptII* is approved by regulatory authorities to use in pDNA development [20].

Antibiotic concentration in the fermentation is necessary to maintain a selection pressure and to ensure the segregation stability of the pDNA containing *nptII* which encodes neomycin phosphotransferase II (NPTII) [21]. In cells harboring pDNA, biomass yield and *µ* were lower and metabolic overflow was higher than that achieved using cells without pDNA. It’s suggested that pDNA yield expected could theoretically be higher than that experimentally obtained by weakening *nptII* expression, which would allow more precursor metabolites and energy, also reduce power needed to be used for plasmid production [22,23]. This metabolic burden is associated mainly with the synthesis of NPTII and not necessarily to the increased plasmid size [22]. Some reports indicate that approximately 20% of the total intracellular protein is the resistance marker and the amount of NPTII that is expressed is much greater than that required for selection and maintenance. In addition, high NPTII specific yield has adverse effects on pDNA downstream process [22,24]. The aminoglycosides and osmotic stress activate *cps* gene clusters expression of *E. coli*. These clusters encode several enzymes involved in the synthesis and translocation of exopolysaccharide and capsular polysaccharide (CPS) layers [25] which show an effect on the absorption of aminoglycosides and could be involved in cell protection. The CPS (generally called endotoxins) is the main contaminant in the production of biological products by *E. coli* [26,27,28].

Kanamycin and temperature induce the synthesis of several proteins, the ones encoded by *cps* cluster and the HSPs, these proteins protect bacterial membrane potential against exposure to kanamycin and can rescue the cell growth [29,30,31]. Scanning electron microscopy (SEM) images of treated samples have been used to observe cell damage and morphological changes with more detail [32]. Some works report the pDNA production by changing the temperature or kanamycin concentration [11,21]. However, the effect of kanamycin concentration as an additional stress factor on pDNA production systems of *Escherichia coli* DH5α has been not evaluated using temperature increase in fed-batch cultures. Thus, the objective of this work was to evaluate the synergic effect of the temperature and the kanamycin concentration on the production of pDNA, NPTII, organic acids (mainly acetate), sc-pDNA and cell morphology in batch and high cell density cultures (HCDC) of *E. coli* containing pVAX1-NH36 plasmid (*E. coli* DH5α-pVAX-NH36).

## 2. Materials and Methods

### 2.1. Bacterial Strain and Plasmid

The host-plasmid combination was *E. coli* DH5α-pVAX-NH36 (3936-bp), which contains a DNA fragment that encodes NH36 antigen of *Leishmania donovani* [33]. pVAX-NH36 contains *nptII* and a pUC origin (pMB1-derived) for high-copy number replication (Invitrogen, 2012). *E. coli* DH5α-pVAX1-NH36 was propagated on LB agar plates with soy peptone and kanamycin (50 µg/mL). A single colony from this plate was used to inoculate a 1000 mL baffled flask with aeration system containing 200 mL of chemically defined mineral medium (CDMM, described below) plus glycerol (12.5 g/L). The shake flask was incubated in an orbital shaker (New Brunswick, USA) carried out at 30 °C, 250 rpm and 0.5 vvm. For cell cryo-protection, 40 mL of glycerol 80% (*w*/*v*) was added when the culture reached an optical density (OD_600nm_) of 8, and aliquots of 10 mL were frozen immediately on dry ice and stored at −70 °C (CDMM seed bank).

### 2.2. Medium and Inoculum

#### 2.2.1. Batch Cultures

The CDMM for *E. coli* DH5α growth (previously modified [8]) had the following composition (g/L): K_2_HPO_4_, 5.8; KH_2_PO_4_. 7.5; antifoam 204, 0.125; (NH_4_)_2_SO_4_, 5.92; MgSO_4_·7H_2_O, 2.3; NaCl, 2; FeCl_3_·6H_2_O, 2.85 mg/L; and 2 mL/L of trace mineral solution (TMS). The TMS contained the following composition (g/L): ZnCl_2_·4H_2_O, 2; CoCl·6H_2_O, 2; Na_2_MoO_4_·2H_2_O, 2; CuCl_2_·2H_2_O, 1.9; H_3_BO_3_, 1.6; MnSO_4_·H_2_O, 1.6; citric acid, 0.6; and CaCl_2_, 1. The fermentations were performed with CDMM plus glycerol and thiamine 25 g/L and 40 µg/mL, respectively.

#### 2.2.2. Fed-Batch Cultures

The CDMM for the initial batch culture had the following composition (g/L): K_2_HPO_4_, 5.8; KH_2_PO_4_, 7.5; antifoam 204 from Sigma-Aldrich (St. Louis, MO, USA), 0.0125; (NH_4_)_2_SO_4_, 2.5; MgSO_4_·7H_2_O, 2.3; NaCl, 2; FeCl_3_·6H_2_O, 22.7 mg/L; and 4 mL/L of TMS. The feeding solution had the following composition (g/L): glycerol, 650; kanamycin, 0.05; thiamine hydrochloride, 0.04; MgSO_4_·7H_2_O, 1.15; TMS, 4 mL/L.

#### 2.2.3. Inoculum

An amount of 10 mL of CDMM cryo-conserved seed bank was inoculated into a 750 mL jar bioreactor containing 200 mL of CDMM plus 12.5 g/L glycerol, 50 µg/mL kanamycin and 40 µg/mL thiamine and carried out at 30 °C, 1200 rpm, 1 vvm without pH control. The culture was used as inoculum (20% *v*/*v*) when an increase of %DO was registered, which meant the complete consumption of glycerol with an OD_600nm_ of 24 (DCW of 9 g/L). This inoculum was used for all experiments.

### 2.3. pDNA Production at Different Temperatures and Kanamycin Concentrations in Batch Culture

#### Experimental Factorial Design 2^2^ with Central Component to Evaluate the Effect of Temperature and Kanamycin Concentration on pDNA and NPTII Production

The effect of temperature and kanamycin concentration on pDNA production was evaluated by using an experimental factorial design 2^2^ with a central component shown in Table 1. This experimental design allowed us to study the effect of each factor on the response variable, as well as the effect of the interactions between factors on the variable with a minimum of experiments. The pDNA production consisted in an initial growth phase to accumulate a high biomass concentration at low temperatures (30–37 °C), followed by a temperature upshift (42–45 °C) to induce a high pDNA replication rate (revised by Jaén et al. [11]). For this reason, the temperature level used were 30 and 42 °C. Likewise, selection marker concentration can drastically affect the plasmid yield and antibiotic concentration levels, therefore, they were selected according to ones reported by Feizollahzadeh et al. [34]. The statistical analysis was performed at 99% confidence level using the software Minitab 17.

In addition, the initial kanamycin concentration of 50 mg/L (concentration commonly used in pDNA production) was evaluated in batch cultures at 30 and 42 °C.

Duplicated fermentations were carried out in a 2 L bioreactor with 1 L of CDMM plus 200 mL of inoculum and performed at 1200 rpm, 1 vvm. The %DO was maintained above 30% air saturation by enriching air with pure oxygen and pH of 7.2 was controlled by automatic addition of 30% NH_4_OH solution throughout the whole fermentation. Samples were taken periodically in the fermentation to determine dry cell weight (DCW) and residual glycerol. A 5 mL sample was taken when the glycerol was totally consumed to determine pDNA yields, purity as % sc-pDNA, organic acids, NPTII yields, and to visualize morphology changes by SEM.

### 2.4. pDNA Production in High-Cell Density Culture (HCDC) with High Kanamycin Concentration

Prior to carrying out the experiments to evaluate the effect of high kanamycin concentration on pDNA production in HCDC, a control glycerol-limited fed-batch culture was carried out in a 2 L bioreactor at 1200 rpm, 1 vvm and pH of 7.2 controlled by automatic titration with 30% NH_4_OH solution. The %DO was maintained above 30% air saturation by enriching air with pure oxygen.

The glycerol-limited fed-batch culture was done as follows: when a batch phase with 25 mL of CDMM cryo-conserved seed bank plus 1 L of CDMM plus kanamycin (25 mg/L) and thiamine (40 µg/mL) at 30 °C reached an OD_600nm_ of 1.3 (0.5 gDCW/L); both pH control and fed-batch phase were started. The fed-batch culture was designed to obtain three growth phases described below at different temperatures. The feeding pump was programmed according to Equation (1)

(1) Ft=μXVeμtYxsSi

where for each growth phase, Ft is the glycerol feeding rate (L/h), *µ* is the desired specific growth rate (h^−1^), X is the initial biomass concentration (gDCW/L), V is the initial volume of media of culture (L), Y_x/s_ is the yield coefficient of biomass-substrate (g/g), and t is the time when the phase began (h). S_i_ is the glycerol concentration in feeding solution (650 g/L) used during all fed-batch phase

Phase A. For cell propagation at 30 °C until the culture reached OD_600nm_ = 21 (8 gDCW/L), F(t) was calculated using a *µ* = 0.27 h^−1^ and Y_x/s_ = 0.35 g/g.

Phase B. When an OD_600nm_ = 21 (8 gDCW/L) was reached during fed-batch phase A, F(t) was adjusted using a *µ* = 0.27 h^−1^ and Y_x/s_ = 0.5 g/g and the temperature increase was started with a heating rate of 0.025 °C/min until 37 °C was reached.

Phase C. When temperature reached 37 °C in phase B, the F(t) was adjusted using a *µ* = 0.15 h^−1^ and Y_x/s_ = 0.5 g/g until an OD_600nm_ = 132 (50 gDCW/L) was reached, the heating rate was maintained at 0.025 °C/min until 42 °C was reached.

The evaluation of the effect of high kanamycin concentration on pDNA production in HCDC was performed using the same strategy of fed-batch culture described above. During fed-batch culture, the kanamycin concentration was increased to 300 mg/L when temperature increase was started. The DCW, residual glycerol, pDNA, organic acids, and NPTII yields were analyzed taking 5 mL of sample in each experiment.

### 2.5. Analytical Methods

#### 2.5.1. DCW and Glycerol Determination

A calibration curve was built previously (gDCW/L = 0.378 × OD_600nm_ provided that OD_600_ < 0.378) to determine the DCW using an appropriate sample dilution. To generate the calibration curve, samples of 10 mL were centrifuged in a Sorval centrifuge at 10,000 rpm and 4 °C for 10 min, washed with distilled water and dried in an oven overnight at 85 °C. The supernatant was used for glycerol quantification using a spectrophotometric method [35].

#### 2.5.2. Plasmid DNA Quantification

pDNA quantification was determined using 300 μg of DCW washed and centrifuged at 10,000 rpm at 4 °C for 10 min. The pellet of each sample was stored at −20 °C for plasmid analysis. The pDNA extraction and purification was carried out according to the manufacturer’s instructions using a QIAprep Spin Miniprep Kit (Quiagen, Hilden, Germany).

The pDNA concentration in purified solution from 300 μg of DCW was measured spectrophotometrically at OD_260nm_ and OD_280nm_ using the Nano-Drop UV spectrophotometer ND-1000 (NanoDrop, Wilmington, DE, USA). To calculate pDNA concentration, the correlation OD_260nm_ = 1 corresponding to a pDNA concentration of 50 mg/L in the purified solution was used.

##### sc-pDNA Quantification

sc-pDNA isoform in purified solution was quantified by electrophoresis using a 0.8% agarose gel in TBE buffer (45 mM Tris HCl, 45 mM H_3_BO_3_, 1 mM EDTA pH 8.0) at 50 V for 90 min and stained with ethidium bromide (5 µg/mL). Total pDNA fractions, including sc-pDNA isoform were estimated using a densitometric analysis of pDNA band intensities in the ImageJ Software version 1.47 and a known amount of a calibrated 1 kb DNA Ladder (New England Biolabs, Ipswich, MA, USA) as a standard.

#### 2.5.3. Organic Acids and NPTII Quantification

Organic acids were analyzed in the supernatants using a HPLC Varian PS-430 (Agilent Technologies., Santa Clara, CA, USA), as detailed elsewhere [36].

Determination of NTPII was done by enzyme-linked immunosorbent assay (ELISA), taking 1 mL of each sample and centrifuged at 10,000 rpm for 10 min at 4 °C to obtain cellular extract. The pellets were re-suspended at same initial volume in extraction buffer (50 mM Tris-HCl from Bio-Rad pH 7.4, 200 mM NaCl from J.T Baker, 15 mM EDTA from Bio-Rad, 100 µM PMSF from Sigma-Aldrich, St. Louis, MO, USA). Samples were sonicated by a cycle of 50% amplitude for 45 s, then shut off for 1 min, repeating the cycle 4 times. A total of 100 µL of cell extracts were added to each well, in duplicate, of the microtiter plate and incubated for 2 h at 37 °C. Followed by three washes with 300 µL of wash buffer (phosphate-buffered saline (PBS) solution plus 0.05% Tween 20 from GE Healthcare; PBS solution contained 137 mM NaCl, 2.7 mM KCl, 10 mM Na_2_HPO_4_, and 2 mM KH_2_PO_4_ from J.T Baker, Phillipsburg, NJ, USA). Then, the wells were blocked with 100 µL of block buffer solution (wash buffer solution plus 3% Skim Milk from Difco) at 37 °C for 1 h and washed five times again. After these washes, 100 µL anti-neomycin phosphotransferase II antibody from rabbit (Merck Millipore, Jaffrey Township, NH, USA), which had been diluted 1:200, was added and incubated for 1 h at 37 °C, and then each well was washed five times. A total of 100 µL Goat Anti-Rabbit IgG, (H + L) HRP conjugate from Merck Millipore (Jaffrey Township, NH, USA), which had been diluted 1:2000, was added and incubated for 1 h at 37 °C. The wells were washed and 100 µL of peroxidase substrate solution was added. To stop the reaction, 100 µL of stop solution (1N H_2_SO_4_) was added to each well, and the absorbance was read at 492 nm in a Multiskan EX from Labsystems (USA). A NPTII protein standard stock solution was obtained (Adgia, Elkhart, IN, USA), dissolved in 20 mM Tris-HCl (Bio-Rad, Hercules, CA, USA) pH 8.0 and stored at −80 °C until use. Working solutions were prepared by dilution of NPTII protein standard stock with carbonate buffer solution containing 0.3 M Na_2_CO_3_ and 0.7 M NaHCO_3_ from J.T Baker (Phillipsburg, NJ, USA), to use it in ELISA method. Six calibration curve solutions (0, 18, 36, 72, 108 and 144 ng/mL) were used to calibrate ELISA method.

#### 2.5.4. Scanning Electronic Microscopy (SEM)

To evaluate the effect of the temperature and kanamycin concentration on the morphology of *E. coli* DH5α-pVAX1-NH36 culture, SEM was performed. For all treatments included in experimental design, 1 mL of culture broth was centrifuged for 10 min at 12,000 rpm and 4 °C. Then, the samples were washed twice with PBS buffer (pH = 7.2) and re-suspended in 1 mL of same buffer. Next, 10 µL of suspension was used to coat a glass slide (1 cm × 1 cm). A fixative was placed on the samples (2.5% glutaraldehyde (*v*/*v*) in PBS) overnight, then washed three times with PBS followed by fixation in 1% osmium tetroxide at 4 °C for 2 h.

The fixed cells were washed three times with PBS. Dehydration of samples was achieved by washing with increasing concentrations of ethyl alcohol (70%, 80%, 90% and 100%, Sigma Aldrich, 200 proof, molecular biology grade). Lastly, all samples were freeze-dried (Tousimis, Samdri-795, Rockville, Maryland), then fixed on carbon tape and sputter-coated with gold particles for 2 min (Denton Vacuum Desk V, Los Angeles, CA, USA).

Images were taken by Scanning Electron Microscope JSM-6510LV (JEOL, Tokyo, Japan).

## 3. Results and Discussion

### 3.1. Statistical Analysis of Experimental Design 2^2^ with a Central Component Used in Batch Culture

The analysis of variance (ANOVA) results for volumetric (pDNA) and specific (Y_pDNA/X_) pDNA yields, pDNA-volumetric productivity (pDNA-VP), volumetric (NPTII) and specific yield (Y_NPTII/X_) NPTII yields are given in Table 2.

Here, ANOVA for all yields manifest that they are highly significant (model *F*-values and very low probability values *p* = 0 for all the response variables). The predicted *R*^2^ are close to the adjusted *R*^2^. This shows that the model fits the data adequately. Usually, for the predictions to be significant, *F*-value and *t*-value should be higher than the low probability *p* values. Values of *p* < 0.001 indicate that the model terms are significant at 99% confidence level.

The ANOVA results for variables imply that the linear positive effects of temperature (*p* < 0.01) and kanamycin concentration (*p* < 0.01) dominate in all treatments studied, having a significant influence on pDNA and NPTII production.

The linear contribution of the temperature to the pDNA production is greater than for the NPTII production. In contrast, the linear contribution of the antibiotic concentration is lower, according to ANOVA, in agreement with the observation that high temperatures increase pDNA replication, but not the production of NPTII.

### 3.2. Effect of Temperature and Initial Kanamycin Concentration on Biomass Concentration Increment (∆X), Growth Rate (µ_max_), and Biomass Yield on Glycerol (Y_X/S_) in Batch Culture

A summary of the results obtained evaluating the effect of temperature and initial kanamycin concentration on pDNA production are shown in Table 3.

**Figure 1:**
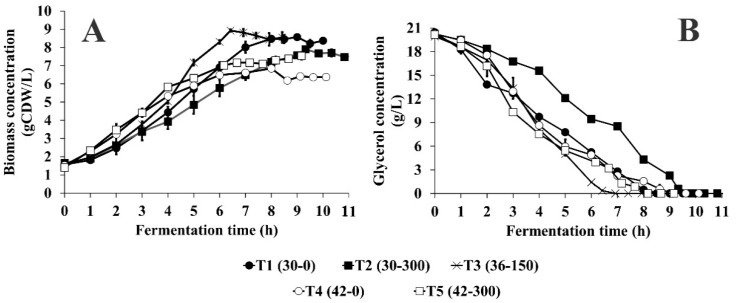
Effect of temperature and initial kanamycin concentration on growth (**A**) and glycerol consumption (**B**) of *E. coli* DH5α-pVAX-NH36 in batch culture. Error bars denote the experimental deviations between duplicate experiments.

The initial glycerol concentration was 20 g/L for all batch cultures in the experimental design (Figure 1B). The maximum Y_X/S_ (0.37 g/g) was obtained in the central point of the experimental design (T_36-150_), carried out at 36 °C with kanamycin concentration of 150 mg/L. This result was to be expected since 36 °C is within the optimum temperature growth range for *E. coli*. It is similar to the ones described previously [37].

Biomass concentration increment was lower when growth temperature was at 30 or 42 °C, consequently the biomass yields on glycerol were also lower (Table 3).

Other works have shown that ∆X and Y_X/S_ are reduced by increasing temperature in pDNA production, however those were evaluated in shake flasks in which the %DO and pH were not controlled [11,12]. At 30 °C, the Y_X/S_ was lower when kanamycin was added (50 and 300 mg/L). Nonetheless, at 42 °C, the Y_X/S_ value significantly increased only when the initial kanamycin concentration was 300 mg/L. All batch cultures showed a first exponential growth phase with a high *µ*_max_ value, then the *µ*_max_ value decreased. The exponential growth phase continued for approximately over two hours before reaching the stationary phase. The highest *µ*_max_ (0.35 h^−1^) was obtained at 42 °C, but this high rate was reached during first 3.5 h, subsequently, it reduced to 0.095 h^−1^ until the stationary phase was reached. At 30 °C, the *µ*_max_ decreased when the initial kanamycin concentration was increased from 0 to 300 mg/L (Figure 1A).

### 3.3. Effect of Temperature and Initial Kanamycin Concentration on Volumetric (pDNA) and Specific (Y_pDNA/X_) Yields, Volumetric Productivity (pDNA-VP) of pDNA and % sc-pDNA in Batch Culture

All pDNA, Y_pDNA/X_ and pDNA-VP high values are required to have a technically and economically viable process of pDNA production. In the central point of experimental design (T_(36-150)_), pDNA value of 57.4 mg/L and Y_pDNA/X_ value of 6.4 mg/g were similar to those reported at 37 °C using the CDMM with glycerol concentration of 50 g/Land kanamycin concentration of 50 mg/L [8]. Yields and pDNA-VP values increased when the temperature was increased agreeing with previous studies using cells containing a pUC temperature inducible replication origin [11,12,38].

When initial kanamycin concentration was increased from 0 to 50 mg/L, no significant effect was detected on these parameters, but, in T_42-300_ with 300 mg/L and 42 °C, these increased more than five times compared with those obtained at 30 °C, regardless of kanamycin concentration. The pDNA replication is highly regulated at temperatures below 37 °C [39]. Thus, the pDNA concentration increase at constant temperature can be attributed to the increase in the antibiotic concentration. When *E. coli* is exposed to β-lactam antibiotics such as ampicillin (also used as a selection marker), the DpiA protein overexpression affects the stability and replication of plasmid by activating DpiAB system in high copy number plasmids such as pVAX1 which contains A+T-rich sequences at replication origin region, and, also, this protein induces SOS response. The kanamycin as a selection marker has molecular advantages for pDNA production, since it does not increase the DpiA protein expression, therefore, it does not activate the DpiAB system [40].

**Figure 2:**
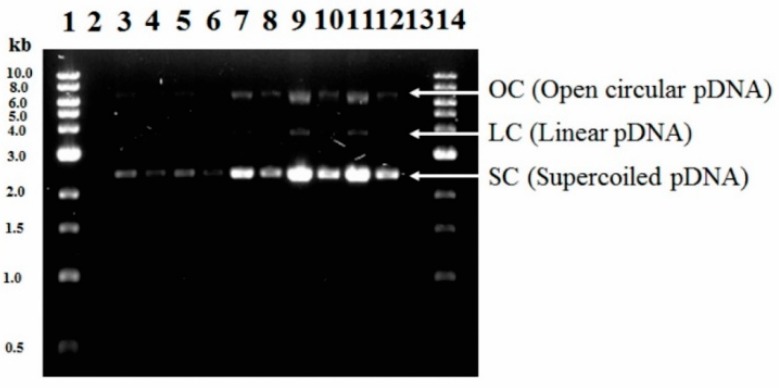
Agarose gel electrophoresis for five different strategies included in experimental design: line 1 and 14, the molecular weight marker (1 kb DNA Ladder); lines 3–4, T_(30-0)_ (1:1 and 1:2); lines 5–6, T_(30-300)_ (1:1 and 1:2); lines 7–8, T_(36-150)_ (1:1 and 1:2); lines 9–10, T_(42-0)_ (1:1 and 1:4); and lines 11–12, T_(42-300)_ (1:1 and 1:4).

The densitometric analysis is presented in Figure 2. The % sc-pDNA were above 90% in all the treatments evaluated in this work which is a requirement to develop DNA vaccines [41,42]. The Federal Drug Administration (FDA) recommends that plasmid DNA vaccines have a content of >80% in the sc-pDNA [43].

### 3.4. Effect of Temperature and Initial Kanamycin Concentration on Metabolic Burden: Concentration (NPTII) and Specific Yield (Y_NPTII/X_) of Neomycin Phosphotransferase II in Batch Culture

The quantification of the NPTII synthesis is important, because it is an indicator of the metabolic burden in *E. coli* DH5α-pVAX1-NH36 fermentation process, likewise, it is considered a contaminant in downstream purification. However, NPTII does not have any of the characteristics associated with allergenic proteins and toxicity in humans. Maximum NPTII concentration (558 mg/L) and Y_NPTII/X_ (61.9 mg/g) were obtained from the central point of experimental design (T_36-150_). These values were 73% and 56% lower at 30 and 42 °C, respectively. The NPTII accumulation was equal at 30 and 42 °C with initial kanamycin concentration of 0 mg/L. However, at 42 °C this accumulation increased by increasing the initial kanamycin concentration. The RpoH protein is an indicator of heat resistance in *E. coli* cultures at high temperature. It regulates the HSPs expression, which confers protection from protein denaturation due to heat. In addition, the exposure to kanamycin causes an increase in RpoH protein levels, which increase heat tolerance [44,45]. At temperatures above 42 °C, the interaction between RpoH and DnaK is reduced due to a conformational change in RpoH, and to DnaK/DnaJ complex chelated by the abundance of heat denatured proteins. This could explain why at 42 °C there is an increase in the synthesis of NPTII as a function of the kanamycin concentration.

### 3.5. Effect of Temperature and Initial Kanamycin Concentration on Overflow Metabolism in Batch Culture

The overflow metabolism products evaluated in this work are shown in Table 4. The acetate accumulation was mainly used as an overflow metabolism indicator. Small amounts of acetate accumulated are in agreement with metabolic characteristics of the strain DH5α used here [46].

It can be observed that high concentrations of antibiotic reduced the production of overflow metabolites (mainly acetate), evidencing a synergistic effect allowing higher pDNA and NPTII. The lower amount of overflow metabolites is a positive effect, because they inhibit the production of biomass and, at higher concentrations, the plasmid replication.

At 30 °C, the acetate concentration decreased 54% when initial kanamycin concentration was increased from 0 to 300 mg/L. On the other hand, in the fermentation carried out at 42 °C and kanamycin concentration of 50 mg/L (T_42-50_) the maximum acetate production reached 4.62 g/L. This value decreased 40 and 74% when the initial kanamycin concentration was 0 and 300 mg/L, respectively. The exposure of *E. coli* to high concentrations of kanamycin evaluated in T_30-300_, T_36-150_, and T_42-300_ resulted in a lower production of overflow metabolism products. This phenomenon could be due to the protein misfolding (involved in the metabolic pathways of glycerol and tricarboxylic acids (TCA) [47], which affect the action and transport mechanisms of aminoglycosides (series of transporters linked to quinones). Requirement of electron transport has been proposed to produce a negative polarity of the transporter due to reduction, this would facilitate a bind to a cationic antibiotic (as kanamycin). The bound aminoglycoside would be driven across the membrane and transferred to ribosomes, the final cellular targets. This process would necessarily limit the quantity of available quinones (or other transporters) for electron flow. Since TCA is linked to bacterial respiration, a block of electron flow would produce a dimishment of the TCA cycle [48]. Likewise, it has been demonstrated that membrane damage caused by aminoglycosides results in a leaky membrane [49], which results in an increase in the re-assimilation of overflow metabolism products, maintaining cell growth and increasing the final pDNA yields. This increase in overflow metabolism products in high temperature fermentation has been reported. Furthermore, the Y_Ac/S_ values, obtained in the present work, were lower and can be explained by the use of glycerol instead of glucose in the fermentations [11,50]. However, the maximum acetate production observed was higher compared to the batch cultures which used glucose at 37 °C [6,51].

### 3.6. Cell Damage and Morphology Changes at Different Temperatures and Initial Kanamycin Concentration in Batch Culture

**Figure 3:**
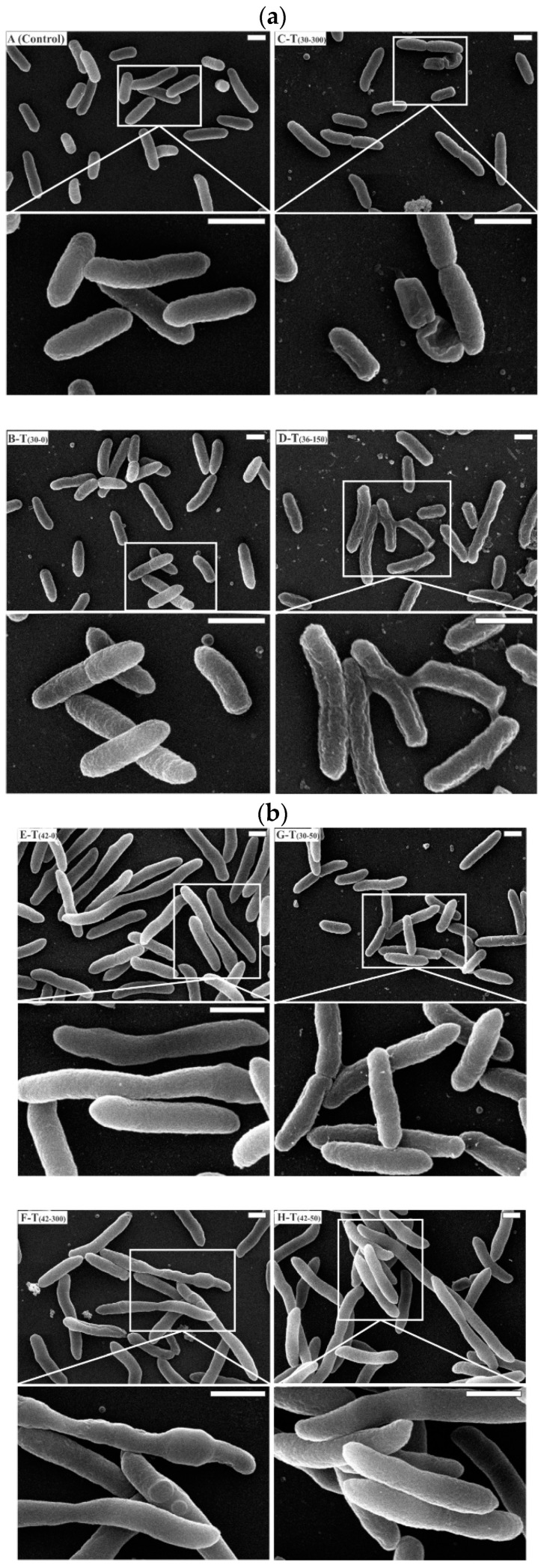
The SEM images by pairs of *Escherichia coli* DH5α samples during experiments carried out in this work. Micrograph above: ×10,000 (▬ 1 µm); below: ×25,000 (▬▬ 1 µm). (**a**) A-Control: control at t = 0 for all treatments were taken at steady-state phase, B-T_(30-0)_, C-T_(30-300)_, D-T_(36-150)_. (**b**) E-T_(42-0)_, F-T_(42-300)_, G-T_(36-50)_, H-T_(42-50)_.

The cell damage and morphological changes of *E. coli* DH5α-pVAX1-NH36 cultures under stress condition by temperature and kanamycin concentration are shown in images taken by SEM (Figure 3a,b).

The image A (Control) in Figure 3a show cells of *E. coli* DH5α of a control sample taken at t = 0, which shows plump rod shapes with an intact membrane, this is the typical morphology. A sample taken at steady-state phase corresponding to T_(30-0)_ condition (B-T_(30-0)_) shows similar characteristics to image A (Control). However, changes were observed in the membrane integrity in all other treatments.

The cells growing with kanamycin concentration of 50 mg/L at 30 and 42 °C are shown in micrograph Figure 3b. *E. coli* cells are rod-shaped and are approximately 800 nm wide and 2.5 µm long in micrograph Figure 3b G–T_(30-50)_ at 30 °C and kanamycin 50 mg/L.

On the other hand, cells grown at 42 °C and kanamycin 50 mg/L in micrograph Figure 3b H-T_(42-50)_ showed a filamentous morphology essentially when cells elongate and replicate their DNA, but do not septate and divide. These morphological characteristics are in agreement with the ones reported by Silva et al. [12]. Cells in micrograph Figure 3a C-T_30-300_ at 30 °C and kanamycin concentration of 300 mg/L showed sinking in the middle section and a shriveled membrane, indicating partial cytoplasm leakage. This damage can be attributed to the high concentration of antibiotic.

The alteration of the surface morphology of *E. coli* cells after treatment with antimicrobial agents has been reported previously [52]. At central composite design treatment of Figure 3a D-T_(36-150)_, the SEM images clearly show limited filamentous morphology and the production of considerable amounts of extracellular CPS. It is important to mention that endotoxins are generally referred as lipopolysaccharides and they are major contaminants in pDNA production by gram negative bacteria, like *E. coli*.

They damage the processes of separation and purification, thus increase the process cost. Consequently, it is proposed not to start cultures of *E. coli* to obtain biomass at 36–37 °C, since it can be observed that CPS production is associated, in this case, with the growth rate. Micrographs Figure 3b E-T_(42-0)_, in Figure 3b, show a filamentous morphology, as evidence shows that nutrient starved cells and oxidative stress activates the filamentous structure [53,54]. In this treatment, an increment in size of four-fold in comparison to control is shown. At 42 °C, it is known that a high-copy number plasmid maintenance and replication imposes a metabolic burden in *E. coli* DH5α, resulting in down-regulation of cell wall biosynthetic genes [55]. In T_(42-300)_ at 42 °C with high kanamycin concentration filamentous morphology and pleomorphism was observed. This can also be observed in *E. coli* when ampicillin is added to growth media [54]. Micrographs Figure 3b F-T_(42-300)_ show the same filamentous morphology. Pleomorphism was also observed, but only under high temperature and kanamycin concentration. Morphological changes including filamentation, has been showed previously in detail for different culture conditions, as starvation and high hydrostatic pressure [54,56]. According to the results provided by the images obtained by SEM, the antibiotic concentration causes greater damage on the cell than the temperature.

### 3.7. Effect of High Kanamycin Concentration on pDNA Production in HCDC

HCDC with, both, high specific yield and productivity of sc-pDNA are required in pDNA production. To achieve these conditions, fed-batch cultures have been developed with different heating rates to increase the temperature and with different feeding strategies to control *µ* [11].

**Figure 4:**
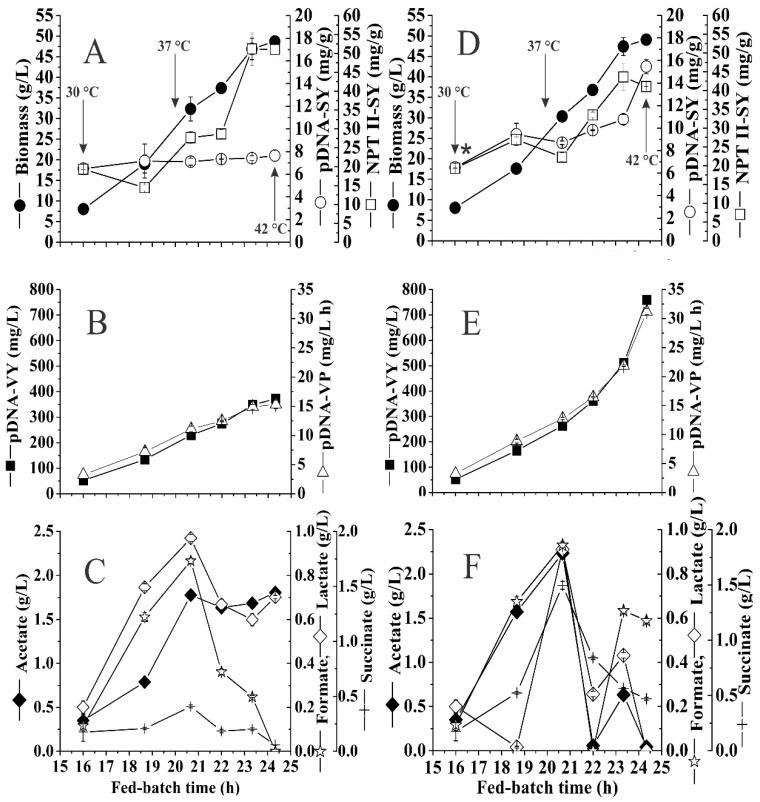
Results of fed-batch cultures of *E. coli* DH5α-pVAX-NH36 with temperature increase from 30 to 42 °C and a heating rate of 0.025 °C/min. Biomass DCW (●); pDNA specific yield (o); NPTII specific yield (□); pDNA volumetric yield (■); pDNA volumetric productivity (∆); acetate (♦), lactate (◊), formate (☆) and succinate concentrations (+). Fed-batch control culture with kanamycin concentration 50 mg/L (**A**–**C**) and fed-batch culture with high kanamycin concentration of 300 mg/L (**D**–**F**), the asterisk represents the addition of kanamycin at 16 h of cultivation. Arrows indicate temperature reached. Error bars denote the experimental standard deviations between duplicate experiments.

In this work, high kanamycin concentration was evaluated as an additional stress factor to the one previously mentioned to increase pDNA production. Figure 4A–C show the results obtained employing the feeding strategy established, a heating rate 0.025 °C/min, and kanamycin concentration of 50 mg/L in the controlled fed-batch culture, which reached 48.75 ± 0.72 gDCW/L with a NPTII specific yield of 51.05 ± 2.37 mg/gDCW and pDNA specific yield of 7.65 ± 0.48 mg/gDCW at 24.3 h of cultivation. In Figure 4B are shown the volumetric yield and productivity being 372.50 ± 17.68 mg/L and 15.31 ± 0.73 mg/L/h, respectively.

Under this condition, the best values obtained for pDNA yields specific and volumetric were similar to those reported by Jaén et al. [11] using the same heating rate (0.025 °C/min) and glucose as carbon source. In our experiment, the Y_pDNA/x_ remained relatively constant when temperature was increased from 30 to 42 °C, during this increase, the organic acid accumulation increased reaching the highest concentration at 37 °C at 20.7 h, but later the value decreased by total consumption of formate and succinate while lactate was partially consumed. Acetate concentration remained almost constant until the end of fed-batch culture, reaching a maximum of 1.8 g/L. This value is low compared to other high-cell-density cultures using DH5α for pDNA production [11,57] but similar to the one obtained with another strain [16,58]. The acetate accumulation, by increasing temperature and higher *µ*, affected the Y_x/s_ value and only had a minor negative effect on plasmid replication [59]. The high concentration of antibiotic (six times more than commonly used) in HCDC does not have statistically significant effects (*p* ˃ 0.05) on biomass, reaching 49.10 ± 1.23 gCDW/L (Figure 4D). However, at the end of the fed-batch phase, important differences were obtained in the specific yields of NPTII and mainly of pDNA being 41.06 ± 0.04 mg/gDCW and 15.47 ± 0.65 mg/gDCW, respectively, which represents a decrease of 20% in the NPTII specific yield and a two-fold increase in the pDNA specific yield, compared to fed-batch control. The important increase in the production of pDNA was after 20.7 h, when the culture reached a temperature of 37 °C and having been exposed to the concentration of 300 mg/L of kanamycin for approximately 4.7 h (Figure 4E). This behavior is evident in terms of volumetric productivity and productivity compared to Figure 4B. The maximum values reached for these two parameters were 759.00 ± 12.73 mg/L and 31.19 ± 0.52 mg/L/h, respectively. Synthesis of overflow metabolism products also showed important differences, the main interest product (acetate), increased 26% (2.24 ± 0.04 g/L) in comparison to the fed-batch control. However, it was almost entirety consumed at the end of the fermentation, which was not observed in the control culture as can be seen in Figure 4C,F.

## 4. Conclusions

In this study, a synergic strategy for pDNA production was evaluated to increase pVAX1-NH36 final yields, using temperature and kanamycin concentration as stress factors. At 42 °C and 0 mg/L, results similar to those reported in the literature were obtained. However, when the combined effect at 42 °C and 300 mg/L was evaluated, an increase of 49%, 35% and 65% in volumetric yield, specific yield and productivity, were obtained. The increase in both pDNA volumetric yield and productivity is mainly due to the increase in the specific yield. The results allow us to provide evidence about the capacity of using high antibiotic concentration to reduce metabolic overflow products (mainly acetate) and NPTII synthesis related to metabolic burden. The combined use of two stress factors as temperature and high kanamycin concentration did not affect the quality of the plasmid produced (sc-pDNA ≥ 90%), being an advantage over the upstream process and its subsequent purification. Finally, based on the micrographs taken to the cultures, it is concluded that starting a culture at 36–37 °C, in the pDNA production process compromises the downstream steps because it involves the synthesis of exopolysaccharides. In addition, the same success was obtained using the high kanamycin concentration (300 mg/L) in HCDC with temperature increase in fed-batch culture. Through this strategy, the pDNA volumetric and specific yields and the volumetric productivity were approximately twice (759.00 ± 12.73 mg/L, 15.47 ± 0.65 mg/gDCW and 31.19 ± 0.52 mg/L/h, respectively) than what were obtained in fed-batch control culture (372.50 ± 17.68 mg/L, 7.65 ± 0.48 mg/gDCW and 15.31 ± 0.73 mg/L/h, respectively). These results suggest that a stress-based process simultaneously caused by high temperature and high kanamycin concentration can be successfully applied to increase the pDNA production by *E. coli* in batch culture. In addition, the same success was obtained using the high kanamycin concentration (300 mg/L) in HCDC with temperature increase in fed-batch culture.

## Figures and Tables

**Table 1:** Experimental factorial design 2^2^ with central component to evaluate the effect of temperature and kanamycin concentration on volumetric (pDNA) and specific (Y_pDNA/X_) pDNA yields, pDNA-volumetric productivity (pDNA-VP), NPTII concentration (NPTII), NPTII specific yield (Y_NPTII/X_) of *Escherichia coli* growing on chemically defined mineral medium (CDMM) with initial glycerol concentration of 20 g/L in batch culture.

Nomenclature	Coded Levels		Real Input Variables Levels	
	X1	X2	Temperature (°C)	Kanamycin (mg/L)
T_(30-0)_	−	−	30	0
T_(30-300)_	−	+	30	300
T_(36-150)_	0	0	36	150
T_(42-0)_	+	−	42	0
T_(42-300)_	+	+	42	300

**Table 2:** Analysis of variance and significance test for pDNA and NPTII yields of *E. coli* growing on CDMM with initial glycerol concentration of 20 g/L in batch culture using experimental factorial design 2^2^ with central component.

Output Variable	Model			Term	*F*-Value	*T*-Value	*p*-Value
	*T*-Value	Predicted R-Square %	Adjusted R-Square %				
pDNA Volumetric Yield	1278.05	99.61	99.86	A	5441.28	73.77	0.000
				B	462.92	21.52	0.000
				AB	271.15	16.47	0.000
				Central point		−14.62	0.000
pDNA Specific Yield	1465.21	99.66	99.88	A	6366.57	79.79	0.000
				B	335.05	18.3	0.000
				AB	139.32	11.8	0.000
				Central point		−22.03	0.000
pDNA Volumetric Productivity	1501.93	99.66	99.88	A	6250.64	79.06	0.000
				B	694.52	26.35	0.000
				AB	551.25	23.48	0.000
				Central point		−3.5	0.025
NPTII Volumetric Yield	647.58	99.23	99.72	A	96.01	9.8	0.001
				B	65.69	8.1	0.001
				AB	91.34	9.56	0.001
				Central point		54.52	0.000
NPTII Specific Yield	988.45	99.49	99.82	A	360.41	18.98	0.000
				B	148.69	12.19	0.000
				AB	143.83	11.99	0.000
				Central point		65.37	0.000

Temperature (A) and kanamycin concentration (B), temperature and kanamycin concentration interaction (AB). The statistical analysis was performed at 99% confidence level using the software Minitab 17.

**Table 3:** Effect of temperature and kanamycin concentration on biomass concentration increment (∆X), biomass yield on glycerol (Y_X/S_), specific growth rate (*µ*), volumetric (pDNA) and specific (Y_pDNA/X_) pDNA yields, pDNA-volumetric productivity (pDNA-VP), NPTII concentration (NPTII), NPTII specific yield (Y_NPTII/X_) and supercoiled isoform of pDNA (sc-pDNA,%) of *E. coli* growing on CDMM with initial glycerol concentration of 20 g/L in batch culture.

Treatment	T_(30-0)_	* T_(30-50)_	T_(30-300)_	T_(36-150)_	T_(42-0)_	* T_(42-50)_	T_(42-300)_
Temperature (°C)	30 °C			36 °C	42 °C		
Kanamycin (mg/L)	0	50	300	150	0	50	300
∆X (g/L)	6.90 ± 0.00	6.43 ± 0.05	6.21 ± 0.20	7.40 ± 0.06	5.32 ± 0.13	5.51 ± 0.26	5.95 ± 0.16
Y_X/S_ (g/g)	0.34 ± 0.01	0.32 ± 0.01	0.31 ± 0.00	0.37 ± 0.00	0.26 ± 0.01	0.27 ± 0.01	0.30 ± 0.01
*µ* (h^−1^)	0.288 ± 0.02	0.247 ± 0.01	0.189 ± 0.01	0.322 ± 0.01	0.353 ± 0.01	0.292 ± 0.03	0.349 ± 0.00
pDNA (mg/L)	27.2 ± 0.76	26.3 ± 0.51	33.9 ± 2.47	57.4 ± 1.71	102.2 ± 1.36	97.6 ± 4.22	151.8 ± 2.55
Y_pDNA/X_ (mg/g)	3.2 ± 0.09	3.1 ± 0.16	4.3 ± 0.34	6.4 ± 0.11	14.9 ± 0.21	12.9 ± 1.93	20.1 ± 0.23
pDNA-VP (mg/L h)	3.2 ± 0.09	3.3 ± 0.22	3.6 ± 0.27	9.0 ± 0.27	11.9 ± 0.16	13.9 ± 1.04	19.7 ± 0.33
NPT II (mg/L)	125 ± 10	152 ± 10	116 ± 11	558 ± 33	127 ± 11	186 ± 10	244 ± 10
Y_NPTII/X_ (mg/g)	14.5 ± 3.27	18.05 ± 2.12	14.6 ± 1.46	61.9 ± 2.67	18.5 ± 1.54	24.6 ± 1.14	32.4 ± 0.41
sc-pDNA (%)	91.58 ± 1.25	92.76 ± 2.76	92.65 ± 1.46	89.98 ± 0.32	89.97 ± 3.6	90.6 ± 4.72	89.85 ± 2.33

These values were taken or calculated when the glycerol was consumed completely, and an increase of %DO was registered by online monitoring and expressed as mean ± standard deviation of two independent fermentations. * These treatments are not included in the experimental design; they were only used to compare with the results obtained in experimental design.

**Table 4:** Effect of temperature and initial kanamycin concentration on maximum organic acids concentration (acetate, lactate, succinate, and formate) of *E. coli* growing on CDMM with initial glycerol concentration of 20 g/L in batch culture.

Treatment	T_(30-0)_	* T_(30-50)_	T_(30-300)_	T_(36-150)_	T_(42-0)_	* T_(42-50)_	T_(42-300)_
Temperature	30 °C			36 °C		42 °C	
Kanamycin (mg/L)	0	50	300	150	0	50	300
Acetate (g/L)	1.07 ± 0.12	0.67 ± 0.06	0.49 ± 0.08	0.59 ± 0.10	2.60 ± 0.11	4.62 ± 0.17	1.18 ± 0.21
Lactate (g/L)	0.06 ± 0.01	0.05 ± 0.00	0.05 ± 0.01	0.10 ± 0.00	0.41 ± 0.04	0.29 ± 0.03	0.23 ± 0.06
Succinate (g/L)	0.17 ± 0.01	0.18 ± 0.02	0.19 ± 0.04	0.20 ± 0.05	0.63 ± 0.07	0.14 ± 0.02	0.39 ± 0.06
Formate (g/L)	0.07 ± 0.00	0.05 ± 0.01	0.11 ± 0.03	0.09 ± 0.00	0.11 ± 0.02	0.05 ± 0.00	0.09 ± 0.01

The results are expressed as mean ± standard deviation of two independent fermentation runs. Samples were taken at stationary-state phase when the glycerol was consumed completely, and an increase of percentage of dissolved oxygen (%DO) was registered by online monitoring (data not shown). * These treatments are not included in the experimental design; they were only used to compare with the results obtained in experimental design.
